# Amnestic MCI patients’ experiences after disclosure of their amyloid PET result in a research context

**DOI:** 10.1186/s13195-017-0321-3

**Published:** 2017-12-02

**Authors:** Gwendolien Vanderschaeghe, Jolien Schaeverbeke, Rose Bruffaerts, Rik Vandenberghe, Kris Dierickx

**Affiliations:** 1 Department of Public Health and Primary Care, Centre for Biomedical Ethics and Law, KU Leuven, Kapucijnenvoer 35 Blok D, Box 7001, 3000 Leuven, Belgium; 2 Department of Neurosciences, Laboratory for Cognitive Neurology, KU Leuven, O&N II, Herestraat 49, Box 1021, 3000 Leuven, Belgium; 3 Alzheimer Research Centre KU Leuven, Leuven research Institute for Neuroscience and Disease, KU Leuven, Leuven, Belgium; 4 Neurology Department, University Hospitals Leuven (UZ Leuven, Campus Gasthuisberg), Leuven, Belgium

**Keywords:** Ethics, Amnestic MCI, MCI due to AD, Research, Qualitative research, Disclosure, Individual research results, Biomarker, Amyloid PET, Belgium

## Abstract

**Background:**

Biomarkers such as amyloid imaging are increasingly used for diagnosis in the early stages of Alzheimer’s disease. Very few studies have examined this from the perspective of the patient. To date, there is only limited evidence about how patients experience and value disclosure in an early disease stage.

**Methods:**

Semistructured interviews were carried out with 38 patients with amnestic mild cognitive impairment as part of an investigator-driven diagnostic trial (EudraCT, 2013-004671-12; registered on 20 June 2014) in which participants could opt to know the binary outcome (positive/negative) result of their amyloid positron emission tomography (PET) scan. Verbatim transcripts of the interviews were evaluated using qualitative content analysis and NVivo 11 software.

**Results:**

Eight of 38 patients received a positive amyloid PET scan result, and the remaining 30 patients received a negative amyloid PET scan result. After disclosure of the result to the patients, we interviewed each patient twice: 2 weeks after disclosure and 6 months after disclosure. Patients had difficulties in repeating the exact words used during disclosure of their amyloid PET scan result by the neurologist; yet, they could recall the core message of the result in their own words. Some patients were confused by the terminology of an amyloid-positive/negative test result. At 6 months, two of eight patients with a positive amyloid PET scan result experienced emotional difficulties (sadness, feeling worried). Three of 30 patients with a negative amyloid PET scan result started to doubt whether they had received the correct result. Patients reported that they experienced advantages after the disclosure, such as information about their health status, the possibility of making practical arrangements, medication, enjoying life more, and a positive impact on relationships. They also reported disadvantages following disclosure, such as having emotional difficulties, feeling worried about when their symptoms might worsen, the risk of a more patronizing attitude by relatives, and the possibility of a wrong diagnosis.

**Conclusions:**

This exploratory study shows that the majority of patients can accurately recall the information received during disclosure. The experienced advantages and disadvantages reported by our patients depended on the outcome of the result (positive or negative) and the interval of the conducted interview (2 weeks or 6 months after amyloid PET disclosure). Discrepancies were found between patients’ expectations according to the interview prior to amyloid PET disclosure (Vanderschaeghe et al. [Neuroethics. 2017;10:281–97]) and their actual experiences after their amyloid PET disclosure.

## Background

In the early, predementia stage of Alzheimer’s disease (AD), clinical, neuropsychological, and magnetic resonance imaging characteristics may not suffice for a reliable AD diagnosis. Especially in the early phases of the disease, biomarkers of the underlying amyloid pathology play an increasingly important role in the recommendations for diagnostic guidelines, in particular in a clinical research setting [1–3]. In most current clinical trials of potentially disease-modifying drugs in AD, biomarker positivity is considered an essential criterion for inclusion of patients.

Several questions remain before amyloid positron emission tomography (PET) can be recommended for application in routine clinical practice in amnestic mild cognitive impairment (aMCI) [4, 5]. These questions relate, among others, to the prediction of the individual timeline of cognitive decline and the psychological consequences of amyloid PET disclosure [6–9]. There is also limited knowledge on how to best disclose amyloid PET scan results to the tested individual [7, 10–12].

A key perspective that has been relatively underrepresented until now is the perspective of the patients themselves [13–15]. One study explored the views of patients and caregivers on amyloid PET imaging [16]. In that study, the views of the 23 caregivers outnumbered the views of the 9 included patients (7 patients with an amyloid-positive PET scan, 2 with an amyloid-negative PET scan) [16]. In addition, the diagnosis in the patient population (*n* = 26) was heterogeneous (e.g., mild cognitive impairment [MCI], atypical presentation of AD dementia, young-onset cognitive impairment) [16]. A number of previous studies have examined the degree to which study participants wish to be informed about the outcomes of tests, but these have been performed mainly in cognitively intact individuals. In an academic memory clinic-based study published in 2015, researchers reported that 45.5% (95 of 209) of cognitively normal participants in a longitudinal aging study were extremely interested in knowing their biomarker result, although this proportion decreased slightly after an education intervention [17]. A review by Bemelmans et al. covered 14 studies of disclosure of genetic AD biomarkers [18]. For example, the Risk Evaluation and Education for Alzheimer’s Disease (REVEAL) study investigated preferences for undergoing genetic apolipoprotein E susceptibility testing for AD in healthy adults who had at least one relative with AD [19, 20]. For amyloid PET, far fewer studies have reported the perspectives of study participants on the disclosure of their results.

A suitable research methodology for gaining insight into patients’ perspectives and experiences is qualitative research [21, 22]. Hence, we conducted an exploratory study with 38 patients with aMCI. Our present study may provide novel qualitative data on how patients with aMCI perceive and experience amyloid PET scan disclosure in a research setting. This study was performed as an optional substudy of a longitudinal observational cohort study about the predictive value of amyloid PET in aMCI. Subjects were offered the possibility of receiving their amyloid PET scan results.

## Methods

### Recruitment

Recruitment took place between June 2015 and June 2016 after approval of the study by the University Hospitals Leuven Ethics Committee. All participants provided written informed consent in accordance with the Declaration of Helsinki.

The study cohort consisted of a consecutive series of 38 patients with aMCI recruited via the memory clinic of the University Hospitals Leuven. The interviews were part of the BioAdaptAD (Biomarker based adaptive development in Alzheimer disease) study, which is an investigator-driven diagnostic trial in aMCI. The primary objective of the BioAdaptAD study (EudraCT, 2013-004671-12) was to evaluate the predictive value of baseline amyloid biomarker measurements for longitudinal change over a 2-year period. When participants met the inclusion criteria (*see* Appendix 1 for all inclusion and exclusion criteria) of the BioAdaptAD study, participants were given the option to participate in the substudy investigating the ethical opportunities and challenges regarding the return of their amyloid PET scan result.

All participants who agreed to volunteer in the substudy had the possibility to be informed of their amyloid PET scan results. Therefore, no protocol was needed to determine who would learn their amyloid PET scan results. This option was favored by the research team for two reasons: first, to respect the individual decision making of the patients, and second, to investigate how many of the participants would choose to learn their amyloid PET scan results. The substudy consisted of three semistructured interviews that were conducted with the aim of getting insight into participants’ motivations and expectations before the disclosure of their results (findings reported by Vanderschaeghe et al. [14]) and participants’ experiences after the disclosure of their results. This paper is focused on the findings of the two interviews conducted 2 weeks and 6 months after the amyloid PET disclosure.

Before the start of the substudy, an informed consent brochure on the general study and the substudy was provided to the participant. This informed consent brochure was based upon the International Conference on Harmonisation (ICH) E6 tripartite guideline of good clinical practice (GCP) [23] and contained background information, objectives, interview process, and participants’ rights. Additional time before the first scheduled interview (before amyloid PET scan disclosure) was provided in which the interviewer orally repeated the content of the informed consent brochure and asked the participants if they had any additional questions. During the two interviews after the amyloid PET scan disclosure, the researcher repeated the participants’ rights and the interview process.

Participants were asked to complete the sociodemographic form and to sign the informed consent form. The latter verified that participants understood the following: that their results and interview recordings were to remain confidential, that their participation was voluntary, and that withdrawal from the substudy would have no impact on their participation in the BioAdaptAD study or on their ability to receive any other medical intervention at the hospital. Participants were informed that the results of this study would be published in a scientific journal and that general findings derived from our interview study would be provided to them after completion of the substudy.

### Diagnostic consultation for amyloid PET disclosure

The disclosure of the amyloid PET scan results was performed by a study physician who was a neurologist (RV or RB). The disclosure happened 2–3 weeks following acquisition of the amyloid PET scan in a semistandardized fashion. First, the physician explained that the visit was a study visit to communicate the amyloid PET result because the person had provided informed consent to participate in the disclosure substudy. Second, the physician explained in general terms what an amyloid PET scan measures and that a scan can show either an increased level of amyloid or a normal level. The physician explained that an increased level (or positive amyloid PET scan result) meant in practical terms that the memory problems were caused by a very early stage of AD and that this also meant that over time there was a high chance that the memory problems would deteriorate further. The physician explained that a normal level (or a negative amyloid PET scan result) meant in practical terms that the memory problems were not due to AD. Third, the physician disclosed the amyloid PET result of the individual patient using the same terminology. In case of a positive amyloid PET scan, the physician emphasized that an amyloid PET scan can already show increased amyloid levels in cognitively intact subjects before clinical symptoms appear. Fourth, in case of a positive amyloid PET scan, the physician advised the participant to start treatment with a cholinesterase inhibitor and then provided the standard explanation regarding the effect and possible side effects. In particular, it was mentioned that a cholinesterase inhibitor could help stabilize the symptoms for a longer period of time, although it could not be predicted at the individual level for how long. Note that the use of a cholinesterase inhibitor in MCI is an off-label use because it is clinically approved for use only in clinically probable AD. In case of a negative amyloid PET scan, the physician informed the patient that this did not mean that the memory problems were discarded as being unimportant; further follow-up was still advisable because memory problems may have causes other than AD. Fifth, the physician asked whether the patient or the patient’s family had any remaining questions. Following this visit, a study visit report was written that mentioned the amyloid PET scan result, the interpretation of the result, the fact that the result was communicated to the patient, and the planned course of action. This report was sent to the patient’s family physician.

### Data collection and analysis

#### Visual assessment of amyloid PET scans

^18^F-florbetaben (NeuraCeq; Piramal Imaging, Berlin, Germany) amyloid PET scans were obtained during a period of 30 minutes between 90 and 120 minutes postinjection using a Siemens Biograph PET/computed tomography scanner (Siemens Healthcare, Erlangen, Germany). This acquisition window was based on the NeuraCeq European Medicines Agency brochure, which recommends the acquisition of a 20-minute PET scan starting approximately 90 minutes after intravenous injections of ^18^F-florbetaben. From 90 minutes postinjection, the tracer concentration reaches a plateau in healthy control subjects and patients with AD, and an acquisition window of 30 minutes can be expected to further increase the signal-to-noise ratio if tolerated by the patient [24]. Visual assessment of summed ^18^F-florbetaben amyloid PET images in subject space was performed by three independent, blinded, and certified (NeuraCeq training provided by Piramal Imaging) readers. According to the NeuraCeq visual assessment guidelines, the following brain regions were assessed for elevated amyloid levels: lateral temporal lobes, frontal lobes, and posterior cingulate/precuneus and parietal lobes. The ^18^F-florbetaben amyloid PET scan was classified as amyloid-positive (+) or amyloid-negative (−) if at least one of these brain regions showed increased amyloid levels. According to the literature, visual assessment of ^18^F-florbetaben amyloid PET scans has a sensitivity of 98% and a specificity of 89% compared with the histopathological standard of truth [25].

#### Interviews and data analysis

The interview guide was developed by GV, KD, and RV and consisted of four main content areas: recall of one’s result, emotional well-being after the disclosure of the result, experienced advantages and disadvantages of knowing one’s amyloid PET result, and feedback of the trial and interviews. These content areas were based on the existing literature and findings derived from our previous study before patients’ amyloid PET disclosures [14]. A pilot study that consisted of the first two interviews was used to evaluate the interview guide [21].

The interviews were recorded on tape after consent of the interviewee was obtained. The verbatim transcripts were analyzed using NVivo 11 software (QSR International, Doncaster, Australia) and via the qualitative conventional content analysis methodology [26–28]. In a first phase, the interviewer (GV) used the pencil-and-paper method as described in the Qualitative Analysis Guide of Leuven [29]. GV coded the interviews at three different times, leaving an interval of a few weeks between coding processes. Leaving interval times and recoding provides the possibility to check whether new meanings can be attributed to the transcripts, increases rigor, and minimizes subjective interpretation [29]. In a second phase, five interviews were separately coded by an independent researcher (KD). In a final phase, a comparison was made between the codes recorded by the interviewer and the independent researcher, and finally consensus upon the codes was reached. Only after consensus regarding the codes were transcripts inserted into the NVivo 11 qualitative analysis software, which provided the researchers with an additional check [29].

For 2 weeks postinterview, saturation for the negative amyloid cases was reached after 23 of 30 interviews. There was no saturation of amyloid-positive cases at both 2 weeks and 6 months postinterview, owing to the limited number of positive cases as part of this clinical trial [30].

For the 6 months postinterview, saturation for the negative amyloid cases was reached after 20 of 27 interviews. Although saturation for the negative cases was reached before we conducted all interviews, we continued the process of the interviews until the deadline of the substudy. The rationale behind the continuation of the interviews was to provide each participant with the same discourse as part of the general clinical study. However, three 6-month postinterviews were not conducted, owing to the difficulty in finding an interview date that was suitable for the patient as well as before the deadline of the substudy. Nonetheless, this did not affect the data, owing to the data saturation mentioned above.

Interviews were conducted in Dutch, with the exception of one patient who preferred the interview to be conducted in his native language, English. In this publication, all transcribed excerpts from the interviews are translated into English.

## Results

### Study population

Sixty-seven patients were invited to participate in the BioAdaptAD study. Twenty-six of them decided not to participate, and three other patients quit the study after the first neuropsychological screening visit. These 26 patients with MCI were not significantly different in terms of age (*t* = −1.67, *p* = 0.10, mean age 73.5 ± 5.4 years, age range 62–82 years), nor were there any differences in gender (chi-square [1] = 0.86, *p* = 0.36, 14 women/12 men) compared with the group of 38 patients with aMCI who decided to participate in the full study. Most of these individuals did not provide a reason or explanation for their decision not to volunteer, but some referred to the burden of caring for a partner or to a lack of motivation. For the three patients who dropped out of the study, the following three reasons were mentioned: a lack of motivation, disagreement of the partner, and an inability to schedule a new appointment that best suited the patient. This resulted in a study population that consisted of 38 patients with aMCI who met the inclusion criteria (Appendix 1) of the BioAdaptAD study [14, 31]. All patients with aMCI agreed to participate in the additional substudy to obtain their amyloid PET scan results.

Eight ^18^F-florbetaben amyloid PET scans were classified as amyloid-positive, and 30 ^18^F-florbetaben amyloid PET scans were classified as amyloid-negative. Table 1 summarizes the demographic information and the participants’ performance on the conventional neuropsychological tests.

**Table 1:** Demographic characteristics, neuropsychological evaluation, and visual assessment read amyloid positron emission tomography scores

Demographic characteristics	Amyloid-positive (*n* = 8)	Amyloid-negative (*n* = 30)	*p* Value
Age, years, mean ± SD (range)	74.8 ± 4.8 (66–81)	69.8 ± 6.6 (55–83)	0.057
Sex, female/male, n	3/5	13/17	–
Years of education, mean ± SD (range)	13.4 ± 3.4 (8–19)	13.1 ± 3.8 (8–22)	0.679
Neuropsychological evaluation	Amyloid-positive, mean ± SD (range)	Amyloid-negative, mean ± SD (range)	
Global Clinical Dementia Rating	0.5	0.5	–
Cornell Scale for Depression in Dementia	1.5 ± 1.3 (0–3)	3.6 ± 3.1 (0–13)	0.066
Mini Mental State Examination (total possible score of 30)	27.0 ± 2.0 (25–30)	28.5 ± 1.2 (25–30)	0.047
Auditory Verbal Learning Test (AVLT) total learning (total possible score of 75)	29.1 ± 4.1 (23–33)	38.1 ± 10.5 (21–62)	0.011
AVLT long-term percentage delayed recall	45.9 ± 47.7 (0–133.3)	65.9 ± 21.9 (11.1–111.1)	0.062

### Recall of individual amyloid PET scan result

Two weeks after disclosure, the amyloid-positive participants had difficulties in repeating the exact words used during disclosure by the neurologist; yet, they were able to recall the core message of the result in their own words. Patients added that they received their results in an early phase, even though this result does not necessarily imply that their memory problems will decline in the near future.

*“Explanation, amyloid tissue in the brain. I don’t know anymore what exactly has been said.”* (66-year-old woman).

Patients who received a negative amyloid PET scan result described that the neurologist clearly communicated the result. They described how the neurologist first reexplained the trial, including the tests that had been done. Some of the patients mentioned the fact that the neurologist had stated that no amyloid plaques were found on the scan or that their current memory complaints were not due to AD. Patients also mentioned that their result or cognitive health status could be different within 5–10 years.

A general finding in both groups was that some patients confused the terminology of a positive/negative amyloid PET scan result. For example, some participants found it confusing that the neurologist spoke of a positive amyloid PET scan result, even though this was interpreted as “bad news” for the patient. Vice versa, the subjects with negative results reported that they had received a positive result. After 6 months, no differences were noted compared with the 2-week postresult interview.

### Emotional aspects associated with amyloid PET scan disclosure

#### Two weeks after disclosure

Overall, the amyloid-positive participants responded that they did not feel surprised by the news, because they already had experienced some memory complaints; yet, receiving bad news is never a pleasant thing. Some individual reactions were, for example, that a participant said he did not allow this news to control his life in a negative way. Another example was a patient who described initially crying after receiving the news but had come to accept it: *“At that time, I cried for a moment, silently. But yes, what can you do about it? Can I now sit down in my sofa and cry the entire day? That is not possible”* (72-year-old woman). Another patient described her emotional difficulties in accepting and coping with the news as she labeled the result a type of “*verdict*” that she has just a few more years in which she would still function well.

The amyloid-negative participants responded mostly with feelings of relief that their memory complaints were not due to an underlying AD pathology. A minority replied in more neutral words by indicating that they would not cheer or celebrate that they had received a negative test result. Other patients reported that their initial reaction was “*mixed*.” On the one hand, they were happy that AD was not detected on the scan. On the other hand, they were wondering what was causing their memory problems. One of them described it as follows: *“I think that if I don’t have Alzheimer’s disease, then I have something else”* (63-year-old woman).

#### Six months after disclosure

Patients who received a positive amyloid PET scan result responded mostly not feeling surprised at the news, owing to memory complaints they had already experienced. However, some differences from their initial feelings 2 weeks after the disclosure were noted. For example, the patient who initially cried, yet accepted the news, described feeling *“unhappy”* after 6 months. This resulted in a mixed feeling. On the one hand, the patient mentioned how she agreed to come to the memory clinic in the first place only because her husband and children had asked her to. On the other hand, the patient explained that it might be good to know the news now, although she found it difficult to know that her symptoms were due to AD when she experienced them as *“not that bad”* and was initially not too concerned about her memory problems. This is in contrast to another patient who was grateful for the news. This result changed his perspective and view on life. Despite his memory complaints, he now felt more self-confident. As he explained it, *“I think good. Yes, I have more self-confidence again.… I feel it in my daily life, in the daily approach with others”* (74-year-old man). One patient described at both postinterviews that she had emotional difficulties in accepting and coping with the news.

No major differences were noted in the patients who received a negative amyloid PET scan result. Most of the patients said they were not thinking of their result anymore. A patient clarified that in the previous interview, he had felt angry about the received result because he was constantly worried and thinking of his memory complaints, which made him angry when the researcher explained his complaints were not due to AD. After 6 months, he described how he could understand his negative result better than at the previous interview 2 weeks after the disclosure of the result.

### Informing others, or not?

#### Two weeks after disclosure

The amyloid-positive participants mostly informed their children and close family members of the news. In most of the cases, the partner of the patient was present at the moment of disclosure. Patients indicated that their partner and children were not surprised about the news, because most of them already noticed the memory complaints of the patient. Two patients who received a positive amyloid PET scan result still had to inform their children because they had not yet had enough time to see their children or had doubts about informing their children out of fear of putting a burden on them.

When patients with a positive amyloid PET scan result were asked whether they had already informed others (friends, neighbors, and so forth), their reactions were twofold:

1. The majority of the patients with a positive amyloid PET scan result indicated that time was too short to inform others, but that they did not have any problems with talking about the news with others. To inform others about their news was perceived as useful for several reasons, listed in Table 2.

**Table 2:** Reasons to inform or not to inform others

Reasons to inform others (by majority of amyloid-positive patients)	Reasons not to inform others (by large group of amyloid-negative patients)
• Other people would know what is going on with them. • Other people can help the patient and take the patient’s situation into account. • Sooner or later, other people will notice what is going on with the patient. • Because it can do the patient well to talk to other people who can understand their problem.	• The decision was made together with the partner before the disclosure of the amyloid PET scan result about whom they would inform once the result was known. • Once you inform others, you have no control of the information. This could result in people who start to perceive the patient differently or that others start to generalize that the patient already has dementia. • Some people do not know how to respond to bad news. In particular, for younger people, it might be difficult to understand the memory complaints of the participant. • The amyloid PET scan result is your personal and thus private information. • It is uninformative news to tell owing to the negative amyloid PET scan result. If they had received a positive test result, they would have informed their inner circle of family and friends.

2. A minority of patients with a positive amyloid PET scan result were not planning to inform others beyond their inner circle of family and friends. For example, a patient described the fear that once you have disclosed the result, there is no control over how people interpret and spread this news to others:

*“And I do know that it is not something that stays with the people but that will be told to others, and before you know it, you get a name glued on you that is much worse than what is going on. Then I think you gradually become shut down from groups you work with, the people you are busy with, and so on. I do not live alone in society. I still live together with a few people around me, and I have a bit of fear that there is too much chatting about that.”* (79-year-old man)

A large group of the amyloid-negative participants said they informed only their partner, children, and some close family members. In addition, they were not planning on informing many others or on making the news public according to the reasons stated in Table 2.

A minority of patients who received a negative amyloid PET scan result had no problems with informing others. For example, a patient stated that she did not care about how others responded to the news. Therefore, she had no problem with talking about her trial participation and her received result.

#### Six months after disclosure

All patients informed their partners and children about their result. The same notion as described in the first follow-up interview, in which some patients were reluctant to inform others about their results (both positive and negative), was noted in the second follow-up interview. For example, a participant who received a positive amyloid PET scan result described how she found it difficult to talk to others about the news out of fear of receiving negative reactions or comments. This resulted in the attitude of hiding her symptoms and received individual research results (IRR):

*“I still find it very difficult to tell it to strangers or to talk about it with people. With the children it is okay, but with other people I still have the feeling I want to hide it as long as possible.”* (66-year-old woman)

Although the previously mentioned citation refers to the fear of negative responses when informing others, no negative reactions of others’ commenting on the participants’ amyloid PET results were reported by our patients (in both positive and negative cases).

### Experienced advantages

#### Two weeks after disclosure

Patients who received a positive amyloid PET scan result mentioned five experienced advantages 2 weeks after the disclosure (*see* Table 3).

**Table 3:** Patients’ experienced advantages 2 weeks and 6 months after amyloid positron emission tomography scan result disclosure

Patients with an amyloid-positive test result	Patients with an amyloid-negative test result
Two weeks after disclosure: 1. More contact with family 2. Early diagnosis 3. Practical arrangements 4. Medication 5. Clarification to know what is going on with their health Six months after disclosure: Similarities: 1. Early diagnosis 2. Medication 3. Clarification to know what is going on with their health 4. Practical arrangements: tricks for memory complaints Newly mentioned advantages: 1. To enjoy life more 2. More and closer follow-up 3. Better for relationship 4. Reassurance/relief Not mentioned after 6 months: 1. More contact with family	Two weeks after disclosure: 1. To know the news as early as possible 2. To resume normal lifestyle 3. To enjoy life more 4. Planning arrangements in the long term 5. To accept memory complaints better 6. Clarification to know what is going on with their health 7. Reassurance/relief Six months after disclosure: Similarities: 1. To know the news as early as possible 2. To resume normal lifestyle 3. Clarification to know what is going on with their health 4. Reassurance/relief 5. To plan ahead/to plan the future Newly mentioned advantages:/ Not mentioned after 6 months: 1. Enjoy life more 2. To accept memory complaints better

1. More contact with family. For example, a patient indicated how he had experienced more contact with his family since he informed them about the result. He had the impression that they wanted to help him, which he perceived as a positive change. However, he implied that this helping attitude should not change into patronizing him.
2. *Early diagnosis*: Early diagnosis was perceived by a minority of patients as an advantage whereby symptoms were still minor and AD was detected at a very early stage.
3. *Ability to make practical arrangements*: Half of the patients who were either thinking of or already had started to make practical arrangements mentioned this as an advantage. Examples given were to move to a smaller place, make financial arrangements, obtain household help, create order regarding practicalities such as developing a good agenda and setting fixed places for objects such as keys, wallet, and so forth. In addition, one patient started to think of an elderly care setting because it takes time to find and arrange a proper place. Yet, he said he was not ready to live in an elderly care setting, because he was still capable of doing most things by himself.
4. *The option to start medication that can possibly delay symptoms*: Although no cure is available, patients described feeling satisfied that some medication was available to stabilize their symptoms.
5. *To know now what is going on with one’s health*: Patients have received a clarification for the symptoms they are experiencing.

A minority of patients with a negative amyloid PET scan experienced no advantages 2 weeks after their IRR disclosure. Because they did not receive *“bad news,”* as they often described it, life just went on as before the disclosure of the result. However, others mentioned several advantages after the IRR disclosure (Table 3):

1. *Getting the news as soon as possible*: Regardless of the outcome of the result, knowing the news as soon as possible, in an early stage, was perceived as an advantage.
2. *Ability to resume a normal lifestyle*: A patient described how before the disclosure of the result, the fear that something might be wrong determined his life. This resulted in doing only the important (practical) things and suspending nonurgent and nonnecessary habits, such as a daily walk and a monthly visit to the theater.
3. *Ability to enjoy life more*: For example, a patient mentioned that before the disclosure, she would always postpone certain activities, such as traveling. However, the negative amyloid PET scan result made her realize that her news was good; yet, other diseases or accidents could always occur. She gained the insight that she needed to do the things she wants to enjoy now without (further) postponing these activities.
4. *Ability to make long-term plans*: As described by a patient, the negative amyloid PET result gave her more time to plan and arrange things for the future in a better way, but without rushing things now. A positive result would have implied that she urgently needed to make, for example, financial arrangements.
5. *Ability to better accept/cope with memory loss and other AD symptoms*: As a patient described it:
*“I have to live with that now [referring to symptoms]. That is not always easy, but I can accept it better now because it is not from dementia, so I can accept it better, and it is easier for me.”* (61-year-old woman)

6. *Knowledge and increased clarity about their health situation*: Before the disclosure, patients were in doubt, not knowing what was going on. The negative amyloid PET scan result provided them with clarification and the opportunity to *“exclude the worst [disease],”* as they would often describe it. For some, to know what was going on resulted in the fact that they stopped comparing their symptoms with, for example, another family member who has AD, because they now knew their symptoms were not due to AD.
7. *Reassurance or relief*: The majority of patients who had received a negative amyloid PET scan result mentioned this advantage owing to their fear of getting AD. As a patient described it:
*“Yes, it’s a relief.… There was a heavy burden, because you think, misery is about to happen and lots of suffering will come to you. Maybe that is over now. Well, you never know, but nobody knows that.”* (68-year-old woman)

#### Six months after disclosure

Six months after the disclosure, we investigated whether patients experienced any advantages of knowing their result. This resulted in several findings, which are displayed in Table 3.

After 6 months, patients who received a positive amyloid PET scan result experienced three similar advantages compared with the interview 2 weeks after the initial disclosure. These advantages were the use of medication, to know what is going on with their health, and the fact that they received the news in an early, still beginning phase. This time, only external aids to deal with their memory complaints were mentioned as practical arrangement, such as writing things down on paper, keeping track of their agenda, and so forth. Patients referred to four new advantages 6 months after the disclosure of their result:

1. Ability to enjoy life more than before the disclosure of the result
2. Regular follow-up and close monitoring of patients’ memory complaints were an advantage of knowing the result.
3. *Better for relationship*: A minority of patients said that the result improved their relationship and led to a better understanding from their partner’s perspective. Their partner stopped complaining about their memory complaints. In addition, one patient said that in the past, he would have never admitted that he had memory problems. Because of the received result, he now accepts and admits he has memory problems, which for him has had a positive impact on his relationship with his wife.
4. *Feeling of reassurance and relief*: For example, a patient said that the result positively influenced his self-confidence. Before the result, he felt in constant doubt.

Patients who received a negative amyloid PET scan result reported similar experience regarding advantages compared with the interview 2 weeks after the initial disclosure (Table 3). These similarities were clarification, resumption of a normal lifestyle, knowing the news as early as possible, and the feeling of relief. To plan the future was mentioned again, but without explicitly referring to planning in the long term as was described in the interview 2 weeks after the initial disclosure.

### Experienced disadvantages

#### Two weeks after disclosure

A minority of patients who received a positive amyloid PET result stated how their awareness about their experienced memory complaints did not result in the feeling of being shocked or surprised after the disclosure of their result. Because of this awareness, they experienced no disadvantages. However, other patients mentioned different experienced disadvantages after the disclosure of the result (Table 4).

**Table 4:** Patients’ experienced disadvantages 2 weeks and 6 months after amyloid positron emission tomography scan result disclosure

Patients with an amyloid-positive test result	Patients with an amyloid-negative test result
Two weeks after disclosure: 1. Emotional difficulties 2. Worse situation than initially expected 3. Patronized by partner/children 4. Question: Will my symptoms become any worse? If so, when? Six months after disclosure: Similarities: 1. Emotional difficulties 2. Question: Will my symptoms become any worse? If so, when? Newly mentioned disadvantages: 1. Driver’s license Not mentioned after 6 months: 1. Worse situation than initially expected 2. Patronized by partner/children	Two weeks after disclosure: 1. No access to all clinical trial data 2. Question: What is causing my memory complaints? Six months after disclosure: Similarities: 1. Question: What is causing my memory complaints? Newly mentioned disadvantages: 1. Wrong diagnosis? Not mentioned after 6 months: 1. No access to all clinical trial data

1. Emotional difficulties coping with the news, such as crying.
2. *Worse situation than initially expected*: For example, a patient described how she initially experienced her memory complaints as minor complaints. Because of the result, she realized her complaints were more severe.
3. *Experience of a patronizing attitude of the patients’ partner or children*: The feeling that others are taking over practical things while patients are still capable of doing things by themselves was mentioned. For example, a patient described how he understood that his children were most likely just trying to help him. Therefore, he mentioned it as a current minimal concern, but as something he wanted to be aware of in case his children would become too patronizing. One of the patients really feared losing her independence and control of her own life. She would find it difficult if her children would start to check the things she did and would take over.
4. The hope that their current memory complaints would remain stable without becoming any worse was mentioned by half of these patients. Yet, after the disclosure of the result, the question “Will my symptoms become worse, and if so, when?” occurred in their minds, although one participant described the difficulty in predicting the time course of cognitive decline:
*“Yes, I hope for the best. It will definitely evolve. I don’t think it will stay like that, but is that within 5 years? Is that already next year? It’s possible that by next year I’m already completely from the map. Is it within 10 years if I’m still alive by that time? I don’t know. As far as I know, there is no fixed rule for that, I think.”* (81-year-old man)

The majority of the patients with negative amyloid PET results experienced no disadvantage 2 weeks after the disclosure of their results, because the majority of these patients perceived their news as *“good news.”* As a patient who received a negative amyloid PET scan explained, *“Disadvantages, no. No. On the contrary, it is good news, so it can only bring advantages”* (69-year-old woman). However, a minority of patients experienced two disadvantages after receiving their negative amyloid PET result (Table 4).

1. A patient described that it was a disadvantage that they received only their amyloid PET scan result, whereas they did not have access to all data about the trial and about all the conducted tests.
2. Although most of the patients with a negative amyloid PET scan result perceived clarity as an advantage of knowing their result, some were left with the question, “What is causing my memory complaints because I still forget a lot?” This unanswered question stayed on these patients’ minds 2 weeks after the disclosure of the negative result. Two patients described it in the following ways:
*“Then I ask myself, there must be something different that they cannot find or where no research has yet been done.”* (73-year-old man)
*“You are happy and still you think, damn, I forget so much. I would better be sick than not being sick.”* (67-year-old man)

For some patients, a positive amyloid PET scan result was considered better than receiving a negative result. A positive result provided them with more clarity than a negative result whereby questions were left unanswered.

#### Six months after disclosure

Half of the group of patients who received a positive amyloid PET scan result experienced no disadvantages 6 months after the disclosure of their result. However, the other half of the group did report some experienced disadvantages (Table 4).

1. Emotional difficulties with accepting and coping with the news remained an experienced disadvantage of knowing their result. One patient described how she started to feel worried. She said it would be better that she would die before her husband would die. For her, the disclosure of a positive amyloid PET scan made her anxious about the future: What will happen to her if she is suddenly alone and her partner is no longer around to help her in case her memory complaints become worse? When asked whether she had discussed this with her partner, she replied that her husband is very caring but not very communicative. He is also very sensitive. Therefore, she found it difficult to express her concern to him without emotionally harming him.
2. A patient referred to the disadvantage of driving the car. She was temporarily not allowed to drive the car, owing to another medical problem. Yet, she was afraid that her positive amyloid PET scan result would turn this into a fixed problem.
3. The question whether their symptoms will become worse remained a perceived disadvantage 6 months after the disclosure.

Two weeks after the disclosure, patients with a positive amyloid PET result described the disadvantage of being treated in a patronizing manner. Six months after the disclosure, patients did not spontaneously mention this as a disadvantage anymore. Patients who initially mentioned this disadvantage 2 weeks after the disclosure were asked to indicate what their current view on this aspect was. They responded that they no longer feared being patronized or that they had thus far not experienced being patronized by others.

Half of the group of patients who received an amyloid-negative PET scan result reported no experienced disadvantages of knowing one’s IRR. The other half described two disadvantages (Table 5). One of these disadvantages was already mentioned at the interview 2 weeks after the disclosure, which was the following question: What is causing the memory complaints? One new disadvantage occurred after 6 months: A minority of patients doubted their result owing to the remaining memory complaints they were experiencing. For example, a patient thought the researcher mistakenly confused her result with the result from another patient. Another patient talked about false-positive and false-negative results;

*“No, it’s always a faint chance that there’s something wrong, a false-negative or something, or maybe for some reason on that day you are better. There’s a faint chance that it could be a wrong diagnosis.”* (66-year-old man)

### Patient feedback on the trial

We asked patients at the 6-month postinterview to evaluate the study and the interviews as part of this trial. Overall, patients responded that everything went smoothly throughout their clinical trial participation. On the basis of reactions from the patients, two recommendations for future trials emerged:

1. A patient said he would change future trials by letting patients be more involved in the design of the study. As he described it:
*“So maybe you should talk to patients first and ask them what they would want to know and then design the study, if you understand what I mean.”* (66-year-old man)
2. A second recommendation was to receive the result in a written report or document that the patient could show to relatives. This document was intended for the patient himself and may not be confused with the written report, which was provided to the patient’s general practitioner.

Most patients mentioned that the interviews were an added value in the trial because they felt it gave them the desired guidance and follow-up. Additionally, they thought it was an opportunity to talk to researchers who understood their complaints about memory loss. A small number of participants described some interview questions as slightly confrontational. They said it was confronting to talk about their memory loss, even though they also added they had a positive experience in the fact that these questions made them think about their health situation. This included all the patients who received a positive amyloid PET scan and most of the patients who received a negative amyloid PET scan. The latter mentioned that in case of a positive amyloid PET scan result, they would have found it beneficial to talk about their “bad news” and to receive some follow-up counseling.

Before the follow-up interviews in our study, a minority of participants who received a negative amyloid PET scan result doubted whether they would continue these interviews, because they had received *“good news,”* as they described it. In addition, the same patients said that these follow-up interviews were not necessary to implement in future clinical trials.

### Regret of their initial choice?

Six months after the disclosure, we asked whether participants would reaffirm their choice to participate in the study and to know their result if they could turn back time. Among the eight patients who received a positive amyloid PET scan result, seven answered that they would make the same decision regarding their trial participation and the option of being informed of their result again. The patient who had mixed feelings 6 months after the disclosure answered that she doubted whether she would make the same decision again. The patient who had emotional difficulties at both follow-up interviews replied that she would make the same decision again, but that it was not easy emotionally to accept and cope with the news.

All patients who received a negative amyloid PET scan result would reaffirm their participation and their option of being informed about their amyloid PET scan result. They clarified that everything went smoothly for them. Overall, they were pleased with the result. For them, this resulted in feelings of reassurance, less doubt, and more clarity about their health situation.

## Discussion

This qualitative report provides important first-hand in-depth information provided by patients with aMCI about how a positive or negative amyloid PET scan result is received over a time course of 2 weeks to 6 months. Because the goal of this study was to explore the patients’ views and experiences on amyloid PET disclosure, we used the methodology of qualitative content analysis and data saturation. Hence, we did not provide a quantitative analysis for two reasons: (1) there are methodological difficulties in quantifying personal views and experiences, and (2) the study was not powered for quantitative statistical analyses. However, the results allow us to entertain additional thoughts on the five findings described below.

### Understanding one’s amyloid PET scan result

Literature on result disclosure often refers to the difficulties study participants have with interpreting and understanding their results. For example, in the field of genetics, it is not uncommon that participants misunderstand their genetic risk disclosure [32, 33]. In the field of AD, the REVEAL study has indicated that asymptomatic adults with a parent who has AD understand that their risk is higher or lower according to their genotype [19]. Our study is in line with the findings of the REVEAL study, because patients with aMCI were able to understand the core message of their result; yet, they often had trouble in using the scientific terminology (positive/negative amyloid PET scan) of their result. Patients with aMCI often used the word *positive* to describe their “good news,” and vice versa for the negative result. Although some patients misused the terminology, most reported that their result was clearly explained, and they could recollect the core message of their result.

The following two recommendations could help to avoid misinterpretation by patients regarding their amyloid PET scan results:

1. Because it is mandatory according to international guidelines of good clinical practice (ICH GCP), the information brochure provided prior to disclosure should clearly explain the possible types of outcome (positive/negative) of the test in a style that is easy to understand by the participant [23]. In addition, it might be useful and lead to less confusion of the terminology of a positive/negative test result for the researcher to provide an example that is comparable to the amyloid PET scan. This recommendation is based on the study conducted by Vanderschaeghe et al. before the amyloid PET disclosure for which the researcher provided a simple example of an alcohol test to clarify the correct use and interpretation of a positive and negative test result [14].
2. In our present study, patients provided feedback after 6 months about the trial and amyloid PET disclosure. This resulted in a practical recommendation mentioned by one of our patients to provide a written document about the received result.

### Emotional risk after one’s amyloid PET disclosure

Reports in the literature describe the diversity of emotional reactions, such as fear, stress, depression, and the possibility of an increased risk for suicidal behavior, as an ethical challenge after the diagnostic disclosure of a dementia diagnosis [34–37]. In our study, most patients with aMCI responded well to their amyloid PET scan result. Among the patients who received a positive amyloid PET scan result, two of eight (25%) described having emotional difficulties (sadness, feeling worried) after 6 months, with no suicidal ideation being reported. Our findings are in line with studies such as the REVEAL study [8, 19] and, more recently with the findings published by Grill et al. [16]. The researchers in the latter study concluded that for some patients and their caregivers, learning amyloid status can cause sadness and despair but also can result in relief and satisfaction [16]. However, the small sample size of our study limits the precision of this given estimate. Therefore, more quantitative studies in larger populations are needed before this particular finding can be generalized.

It is possible that the risks of developing suicidal behavior did not occur in our study, owing to the following two study limitations: (1) Only eight patients were amyloid-positive, and (2) the interviews were conducted within a time frame of 6 months after the disclosure of the IRR. Literature recommends that a longer time frame for follow-up is necessary to generalize a particular finding [8, 38]. For example, in their study about predictive testing for Huntington’s disease, Timman et al. stated that psychological outcomes 7–10 years after predictive testing are worse than in the 2–3 years after testing [38]. The rationale behind the longer follow-up would be that individuals are closer to the likely age of disease onset, which could lead to more distress levels [38]. Although 7–10 years is long in the case of patients with aMCI, we do recommend longer follow-up for at least several years.

### Patient’ reactions toward their amyloid PET disclosure versus their expectations

In our study, a majority of patients with aMCI who tested positive reported not feeling surprised about their positive amyloid PET scan result, and these patients explained how the result was a confirmation of their experienced memory complaints. This confirms that positive test results are not always perceived as overwhelming if the test results match patients’ expectations [7].

The overall majority of patients who received a negative amyloid PET scan result were happy with their “good news” of being amyloid-negative. However, a minority (3 of 30) of the amyloid-negative patients reported 6 months after the IRR disclosure that they had started to doubt whether they had received the correct test result. Owing to the limited study population of amyloid-negative cases in Grill et al.’s study [16], our study findings establish the “potential dichotomy” (between the perception of amyloid-negative as “good news” or as “clinical uncertainty”) and the need for further research with more amyloid-negative patients as described by Grill et al. [16]. Possible explanations for these different reactions within the amyloid-negative patients are as follows:

1. Patients who received a negative amyloid PET scan result most likely expected to have received a positive test result owing to their experienced memory complaints. After 6 months, the continued experience of their memory complaints led them to doubt their result. Some patients even concluded that a positive test result would have been better than their received negative result. This positive result would probably better match their experienced memory complaints.
2. Another explanation is derived from the study conducted by Linnenbringer et al. [39], who concluded that among participants (healthy adults with at least one relative with AD) who did accurately recall their risk information, nearly one-third perceived their personal risk to develop AD to be higher than the actual communicated risk by the researcher [39]. This finding highlights that participants may accurately understand their received information but may still perceive it slightly differently, depending on, for example, their personal coping strategy and experienced memory complaints in our study population [39–41].

### Advantages and disadvantages of knowing one’s result

Grill et al. reported several advantages and disadvantages of amyloid imaging from patients’ and caregivers’ points of view [16]. One example is the advantage of making informed care decisions and the benefit that the patient now has a diagnosis [16]. Our study population described several pros and cons of knowing one’s IRR, which are in line with, yet cannot be limited to, the described advantages and disadvantages in the study by Grill et al. More importantly, we noticed in our study four modifiers of the given pros and cons as described by our patients. These modifiers are described in the paragraphs below.

First, the outcome of the result—a positive or negative amyloid PET scan result—had an impact on the experienced (dis)advantages. For example, to start medication and to have more contact with family members were mentioned as two advantages (2 weeks after the disclosure) of patients with a positive amyloid PET scan result, but this was not reported by the patients who received a negative amyloid PET scan result.

Second, the time interval of the conducted interviews—2 weeks or 6 months after the disclosure of one’s result—also had an impact on the experienced advantages and disadvantages. For example, a positive impact on the relationship with their partner was mentioned as an advantage at the interview 6 months after the disclosure, but it was not mentioned 2 weeks after the disclosure (with patients who received a positive amyloid PET scan result). Vice versa, patronizing was perceived as a disadvantage 2 weeks after amyloid PET disclosure in participants with a positive amyloid PET scan result, but this was not mentioned again at 6 months. When this issue was readdressed to the patients who mentioned this disadvantage after 2 weeks, they replied they did not (or no longer) experience this issue. Possible explanations are, for example, the initial reactions or coping strategies of some partners and children to dealing with the received information. It is possible that some family members misinterpreted the positive test result as meaning that the patient already had dementia [28].

Third, differences were noted between patients’ expectations before the disclosure of their amyloid PET result and their concrete experiences after the disclosure. For example, making practical arrangements for the future, and in particular the request for euthanasia, was the advantage of knowing one’s result in the interview study that was most frequently mentioned by the patients with aMCI before their amyloid PET disclosure [14]. Results of our present study reveal how arranging for the future was still mentioned but experienced less as a disadvantage of knowing their IRR. In addition, most patients with aMCI no longer have a sense of urgency to make certain arrangements, owing to their negative amyloid PET scan result. The study of Gooding et al. about genetic susceptibility testing for AD stated that planning for the future is important for all elderly people, not excluding people who learned they were not at increased genetic risk for AD [42]. The same notion is applicable to our study in patients with aMCI with a negative result on the amyloid PET scan.

Fourth, our findings reveal how patients’ experiences may not be limited to the expected risks and benefits as described in general literature on amyloid PET disclosure [7, 13, 43, 44] and IRR disclosure [45–47], and in the information brochure of the clinical trial provided according to the ICH GCP guidelines [23].

### Family support and family pressure

Family support is an important aspect when patients volunteer to participate in a clinical trial and opt for the disclosure of their amyloid PET result. Our findings show how patients mostly felt supported by their family members. However, after 6 months, one specific patient who received a positive amyloid PET scan result described her doubts about participating and opting for her IRR disclosure. This patient talked about mixed feelings; it was clear that she would not have participated and come to the memory clinic in the first place if her husband and children had not asked her to do so. Although it might be advantageous to know this news in an early stage, the option of being informed of the result should be made by the patient who is still capable and autonomous enough to make this personal decision, but without the influence of family pressure. This specific case indicates how there is a blurry line between support and pressure and how difficult it may be for external people, such as researchers, to distinguish whether the study participant is supported or pressured by family members. In addition, what study participants may perceive as family support before disclosure can be interpreted differently after disclosure of the result and depending on the outcome of the conducted test(s).

### Limitations

The strength of this qualitative research is the in-depth, face-to-face interviews with patients because this method provided us with the opportunity to clarify and check the opinions of the interviewees. We have to acknowledge several limitations of this exploratory study. A first limitation is that participants are always embedded in a certain cultural or societal setting, which can be reflected in the results. The views expressed here are based upon a small population of patients with aMCI in Belgium who were recruited via the UZ Leuven Memory Clinic, and the study was based upon eight patients who received a positive amyloid test result. The total number of amyloid-positive MCI cases was relatively low (8 of 38), which may be due to the fact that patients with aMCI were recruited regardless of the duration of prior follow-up. Patients who had converted to AD in the months and years following a diagnosis of aMCI would not be eligible for the present study. As a consequence, the sample may have been enriched for non-AD causes of aMCI. Different findings can occur when investigation is done in a different country and when recruiting a different population, such as patients with subjective memory complaints, patients with minor memory complaints who have not been to the memory clinic to receive medical advice, and healthy adults being evaluated for preclinical AD. Last, the quotations used in this paper were translated into English, whereby some of the participants’ nuances may be lost. We do believe that the findings are of importance beyond the context of this research and can be of use for future studies.

## Conclusions

The findings of the present study provide insight into patients’ opinions and experiences that may inform the design of future quantitative larger-scale studies of amyloid PET disclosure. Furthermore, additional research should incorporate longer duration of follow-up interviews after amyloid PET disclosure. This would allow an exploration of how the emotional reactions of patients change over time, as well as how patients with a positive amyloid PET scan result cope with cognitive deterioration.

## Appendix 1

**Table 5:** Inclusion and exclusion criteria of the BioAdaptAD study

Inclusion criteria	Exclusion criteria
Patients must meet all of the following inclusion criteria to participate in the study: • Patient is male or female and between ≥ 55 and ≤ 85 years of age on the day of signing the consent form. • If female, patient is not of reproductive potential (2 years postmenopausal or surgically sterile). • Patient has a subjective memory concern as reported by subject, study partner, or clinician. • Patient has an abnormal memory function documented by scoring below the • Education-adjusted ranges on the Auditory Verbal Learning Test total learning or delayed recall percentage ≥ −1 SD. • Patient has a global Clinical Dementia Rating of 0 or 0.5. • In the opinion of the investigator, the patient is in stable medical condition and willing and able to perform study procedures. • Patient has at least 6 years of education or work history sufficient, in the investigator’s opinion, to exclude intellectual disability.	Patients will be ineligible for participation in this study if they meet any of the following exclusion criteria: • Patient has a history or current evidence of a neurological disorder that, in the opinion of the primary investigator, may contribute to the subject’s cognitive impairment, including but not limited to: – Large-vessel stroke – Epilepsy – Parkinson’s disease – Progressive supranuclear palsy – Huntington’s disease – Amyotrophic lateral sclerosis – Multiple sclerosis – Central nervous system infection – Significant head trauma with loss of consciousness – Normal-pressure hydrocephalus • Patient has a history of large-vessel stroke or evidence of a large-vessel infarction or other focal lesions seen on baseline magnetic resonance imaging (MRI) scan that may contribute to the cause of the memory impairment in the opinion of the investigator. • Patient has received an examination over the past year involving > 10 mSv of ionizing radiation • Patient has a history within 6 months prior to screening visit or current evidence of a psychotic disorder or untreated major depressive disorder. • Patient has a history of malignancy ≤ 5 years prior to signing informed consent, except for patients who have undergone potentially curative therapy with no evidence of recurrence for 1 year and who are deemed at low risk for recurrence by her/his treating physician. • Patient has a history or current evidence of any potentially known clinically significant condition therapy, laboratory abnormality, or other circumstance that, in the opinion of the investigator, might confound the results of the study or interfere with the patient’s participation for the full duration of the study, such that it is not in the best interest of the patient to participate. • Patient currently uses specific psychoactive medications (e.g., neuroleptics, chronic anxiolytics, tricyclic antidepressants, antiepileptics, anticholinergics). Stable-dose trazodone, mirtazapine, or low-dose benzodiazepines prescribed for mild insomnia are allowed. • Patient currently uses antithrombotics, with the exception of acetylsalicylic acid (exclusionary for lumbar puncture). • Patient has a history of alcohol or substance abuse or dependence within the past 2 years (based on criteria of the *Diagnostic and Statistical Manual of Mental Disorders, Fourth Edition*). • Patient is currently participating or has participated in a study with an investigational compound or neuropsychological measures within 30 days of signing informed consent. • Subject has any magnetizable metal prostheses, implants, or foreign objects that could pose a hazard during MRI scanning.

## Abbreviations

AD: Alzheimer’s disease
aMCI: Amnestic mild cognitive impairment
AVLT: Auditory Verbal Learning Test
BioAdaptAD: Biomarker based adaptive development in Alzheimer disease
ICH GCP: International Conference on Harmonisation E6 tripartite guideline of good clinical practice
IRR: Individual research results
MCI: Mild cognitive impairment
MRI: Magnetic resonance imaging
PET: Positron emission tomography
REVEAL: Risk Evaluation and Education for Alzheimer’s Disease study
